# Application of attentional bias modification to reduce attentional bias and emotional reactivity to stress in mildly depressed individuals

**DOI:** 10.3389/fpsyg.2023.1273512

**Published:** 2023-10-27

**Authors:** Soojung Baek, SoSeo Ha, Jang-Han Lee

**Affiliations:** ^1^Department of Educational Affairs, Anyang Juvenile Detention Center, Anyang, Republic of Korea; ^2^Department of Psychology, Chung-Ang University, Seoul, Republic of Korea

**Keywords:** depression, attentional bias modification, emotional reactivity, eye-movement, eye-tracking

## Abstract

This study aims to verify the effectiveness of attentional bias modification (ABM) in reducing attentional bias related to depression, particularly in the later stages of attention as a pattern of difficulty in disengagement from depression-relevant stimuli, and to assess its effects on emotional reactivity to stress. A total of 78 participants were separated into four groups based on their levels of depression (minimal and mild) and the types of ABM. The positive ABM (pABM) trained participants to disengage their attention from depression-relevant stimuli and directed their attention toward more positive stimuli, whereas the neutral ABM (nABM) was designed to have no effect. The participants underwent a free-viewing task by eye tracker both before and after ABM to observe changes in attentional bias. Subsequently, they reported their emotional response after a stress-inducing task. The group of mildly depressed participants receiving pABM showed significantly less attention to depression-relevant negative affective stimuli and reported significantly decreased negative emotional reactivity to stress compared to the other groups. pABM had an effect on decreasing difficulty in disengaging from depression-relevant negative affective words (DW). However, it did not increase the dwell time on positive affective words (PW) in the current study. This might be due to the short duration of the application of ABM. The current study conducted ABM twice in 1 day, and this might not be enough to increase the dwell time on PA. This study verified that the ABM effectively decreased the attentional bias of depression and its relevant symptom, emotional reactivity to stress.

## 1. Introduction

Depressed people have a distinctive pattern of attentional bias in that they have difficulty disengaging from depression-related negative affective stimuli while rarely attending to positive affective stimuli (Koster et al., 2005; Bodenschatz et al., 2019; Klawohn et al., 2020). This attentional bias develops and exacerbates depressive symptoms as it facilitates remembering negative information and overestimating the affective content of negative or neutral information more than it actually is (Joormann and Gotlib, 2007; Grahek et al., 2018). The negatively biased information processing resulting from attentional bias causes emotional reactivity, which is typical of depression, and the causal relationship between the two is believed to result in vulnerability to depression (Beevers et al., 2015).

Emotional reactivity in depression refers to the attenuated positive emotional response to positive events and intensified negative emotional response under stressful circumstances (Clark et al., 1994; Joormann and Gotlib, 2007; McFarland and Klein, 2009; Osinsky et al., 2012; Thoern et al., 2016). Biased attention gives rise to negative emotional reactivity by influencing the processing of negative affective stimuli, and this potentiated negative emotion aggravates depression as it, in turn, interacts with information processing (Beck, 1979; Lemoult and Gotlib, 2019). In addition, the attenuated positive emotion contributes to the abnormal emotional responses of depression, including anhedonia, apathy, and psychomotor delay, because it induces a lack of appetitive motivation (Clark et al., 1994; Hill et al., 2019).

Cognitive therapeutic approaches have been used to reduce the development and recurrence of depression by decreasing emotional reactivity and changing maladaptive cognitive styles. However, these approaches have the limitation of putting patients at a risk of recurrence as they do not address attentional bias (Joormann and Gotlib, 2007; Spinhoven et al., 2018). Even after negative thoughts are modified, attentional bias remains stable and intensifies negative emotional reactivity; therefore, attentional bias should be modified to prevent the recurrence of depression (Linville, 1996; Joormann, 2004; Gotlib and Joormann, 2010).

In this context, attentional bias modification (ABM) that covers the limitation of cognitive therapies, a computerized cognitive behavior modification technique that changes negatively biased attention into positive attentional bias, has been suggested as an alternative therapeutic approach (Mogoaşe et al., 2014). Using experimental paradigms to measure attention, such as the dot-probe task, the visual search task, and the spatial cueing task, ABM repeatedly trains participants to have a more appropriate attentional pattern. It intentionally places a target probe, which they must detect, in the location of the positive or neutral affective stimulus, causing participants to direct more attention to positive affective stimuli than negative affective stimuli (Lazarov et al., 2018; Li et al., 2023). Additionally, some studies found that ABM redirected toward neutral stimuli rather than negative stimuli enhances the ability to disengage from negative attentional biases (Yang et al., 2015; Klawohn et al., 2020).

The therapeutic efficacy of ABM, which decreases emotional symptoms by conducting repetitive training of the attentional pattern, stems from the emotion that is processed at an unconscious level and suggests that the behavioral response at the conscious level can be modified by the implicit manipulation of attentional bias (Maoz et al., 2013; De Voogd et al., 2016). For example, training for searching positive stimuli among negative distractors alleviated anxiety symptoms as well as changed the brain activation for emotional stimuli (Waters et al., 2019). In addition, other studies have reported positive results indicating that ABM prevented the development of depression in mildly depressed individuals and relieved depressive symptoms in general (Li et al., 2016; Yang et al., 2016). However, these studies simply suggested a preventive role of ABM or highlighted relieved depressive symptoms, in general, rather than exploring the consecutive effects of attentional bias and its relevant symptoms.

Theories of visual processing have divided attention into two distinct mechanisms: extracting physical information from visual input (early stage of attention) and generating behavioral responses by evaluating visual information (later stage of attention) (Hollenstein et al., 2012). Regarding the attentional processing of word stimuli, the analysis of the physical characteristics of words takes place in the early stage of attention (from 0 to 200 ms) while the analysis of the content of the words arises in the later stage of attention (after 200 ms) (Hollenstein et al., 2012). Previous studies were found to be inconsistent regarding the stage of attention affected by ABM. It has been suggested that, although ABM is intended to modify the attentional bias in early attention (Sears et al., 2011), its effects are primarily focused on enhancing general attentional control (Bar-Haim, 2010; Sallard et al., 2018; Li et al., 2023). For example, ABM training in depression altered the functional connectivity of attentional network, including the anterior cingulate cortex and the middle frontal gyrus, a region of ventral lateral PFC (Beevers et al., 2015; Li et al., 2016).

The changes in attention resulting from ABM should be carefully monitored when manipulated in the study because both independent and dependent variables can be affected. Most ABM studies adopt experimental paradigms, such as the dot-probe task, the visual search task, or the spatial cueing task, that direct attention by habituating a certain form of behavioral response in a repeated manner (Baert et al., 2010; Kuckertz and Amir, 2015). However, it has been reported that measuring attentional bias by a task such as dot-probe has low test-retest reliability (Schmukle, 2005; MacLeod et al., 2019; McNally, 2019). If the changes in attention resulting from ABM are measured in a similar way, the result could be contaminated by habituation to the ABM task. Thus, it might be effective to measure attention through direct ABM assessment rather than requiring a behavioral response, and measuring attention by eye tracker is useful for this purpose. Eye tracking provides accurate and objective evidence of the attentional process because it directly measures eye-movement patterns in response to stimuli without requiring any response (Duchowski, 2002; Christiansen et al., 2015). In other words, using eye-tracking technique can help determine which stage of attention processing is affected by ABM (Sears et al., 2019).

The current study aims to investigate whether ABM can reduce attentional bias in depression, which occurs in the later stages of attention. To this end, the study employed two types of ABM interventions (positive and neutral) and a free-viewing task that provides a real-time eye-gaze to record participants' attentional bias before and after ABM. Considering that an ABM intervention improves the ability to attentional disengagement from negative stimuli, we hypothesized that the mildly depressed group with ABM would give significantly less attention to depression-relevant negative stimuli and decreased emotional reactivity to stress after ABM.

## 2. Materials and methods

### 2.1. Participants

**Figure 1:**
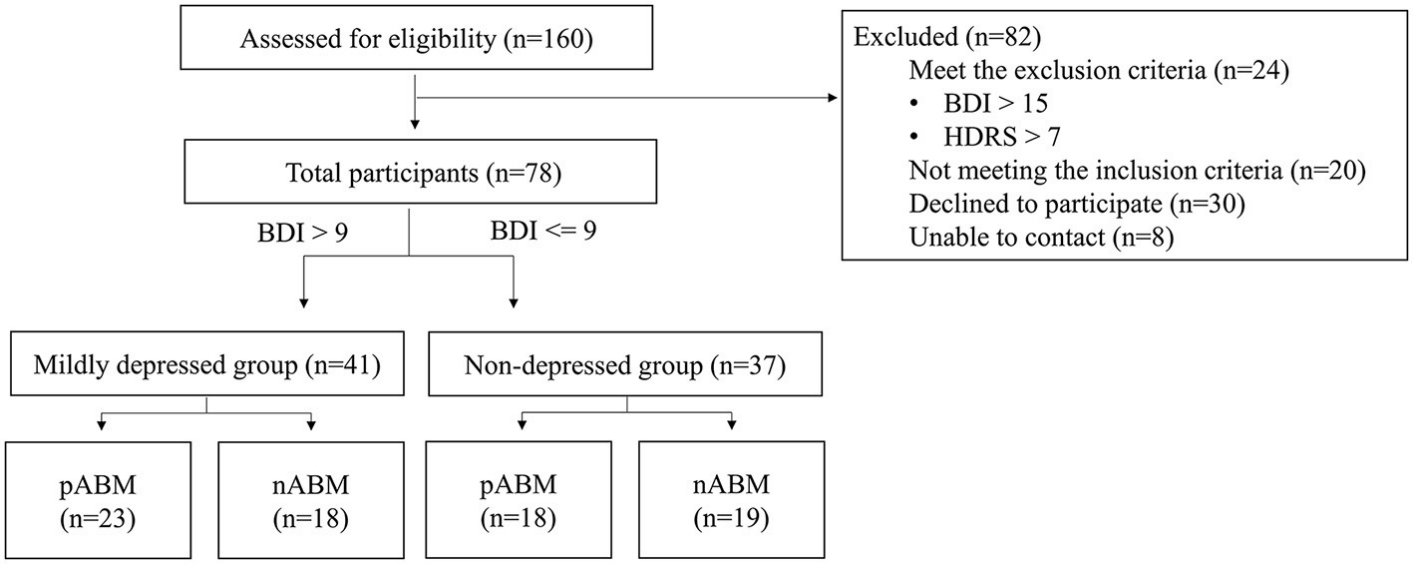
A diagram illustrating the flow of participants through the study.

The Beck Depression Inventory-II (BDI-II: Beck et al., 1996) test and the Hamilton Depression Rating Scale (HDRS; Hamilton, 1960) for pre-screening were completed by 160 undergraduate students. This study included 86 students who underwent screening. According to the findings of a previous study that ABM would have an effect on those suffering from mild depression (Baert et al., 2010), participants with a BDI score ranging from 0 to 9 (*n* = 43) were assigned to the non-depression group, and participants with BDI score ranging from 10 to 15 (*n* = 35) were assigned to the mildly depressed group. As can be seen in Figure 1, HDRS was used to control anxious depression, and the participants whose anxiety/somatization factor scores were higher than 7 points were excluded (Cleary and Guy, 1977).

### 2.2. Self-questionnaires

#### 2.2.1. Beck depression inventory-II

The level of depression was measured by the Beck Depression Inventory-II (Beck et al., 1996) test. This study used the Korean version of BDI-II (Kim et al., 2007) which consisted of 21 items describing physical and cognitive symptoms and was rated on a 4-point scale, ranging from 0 to 3. The cut-off scores for each level of depression were as follows: minimal (0–13), mild (14–19), moderate (20–28), and severe (29–63). In this study, Cronbach's α of the Korean version of BDI-II was 0.75.

#### 2.2.2. Hamilton depression rating scale

The comorbidity of depression and anxiety was controlled by the anxiety/somatization factor items of the Hamilton Depression Rating Scale (Hamilton, 1960). This test consists of a total of 17 items divided among four factors, and the anxiety/somatization factor includes six items. Items describing psychic anxiety, somatic anxiety, and hypochondriasis were rated from 0 to 3 and items describing gastrointestinal problems, general somatic symptoms, and insights were rated from 0 to 2. This study used the Korean version of the HDRS (Yi et al., 2005) and Cronbach's α was 0.64.

#### 2.2.3. The positive affect and negative affect schedule

This study used the Positive and Negative Affect Schedule (PANAS) to measure emotional reactivity to stress (Watson et al., 1988). The PANAS consists of two 10-item subscales: positive affect (PA) and negative affect (NA), rated on a 5-point scale from 1 (not at all) to 5 (extreme). The total score ranges from 10 to 50 for positive and negative affect, respectively, and the score is proportionate to the positive and negative affect. The Korean version of the PANAS (Lee et al., 2003) was used in this study, and Cronbach's α for the PA subscale was 0.88 and the NA subscale was 0.85.

### 2.3. Experimental stimuli

Emotional words from the Korean Emotion Vocabulary List were used as affective stimuli in ABM and the eye-tracking task (Sohn et al., 2012). The word list categorizes emotional words by the feeling generated, and the list was validated by providing the valence and arousal of each word. This study extracted words expressing a single emotion, and then, 13 graduate students majoring in clinical psychology re-rated the valence, arousal, and depression relevance of the words with a 7-point scale. The word stimuli were then categorized as depression-relevant negative affective words (DW), neutral affective words (NeuW), and positive affective words (PW; see Table 1).

**Table 1:** Mean (SD) for depression relevance, valence, and arousal of each type of stimuli.

**Types of stimuli**	**Depression relevance**	**Valence**	**Arousal**
DW	5.51 (0.49)	1.92 (0.30)	4.16 (0.50)
NeuW	3.05 (0.54)	3.63 (0.37)	3.56 (0.58)
PW	1.46 (0.21)	6.39 (0.31)	4.57 (0.40)

DW, depression-relevant negative affective words; NeuW, neutral affective words; PW, positive affective words.

#### 2.3.1. Free-viewing task

**Figure 2:**
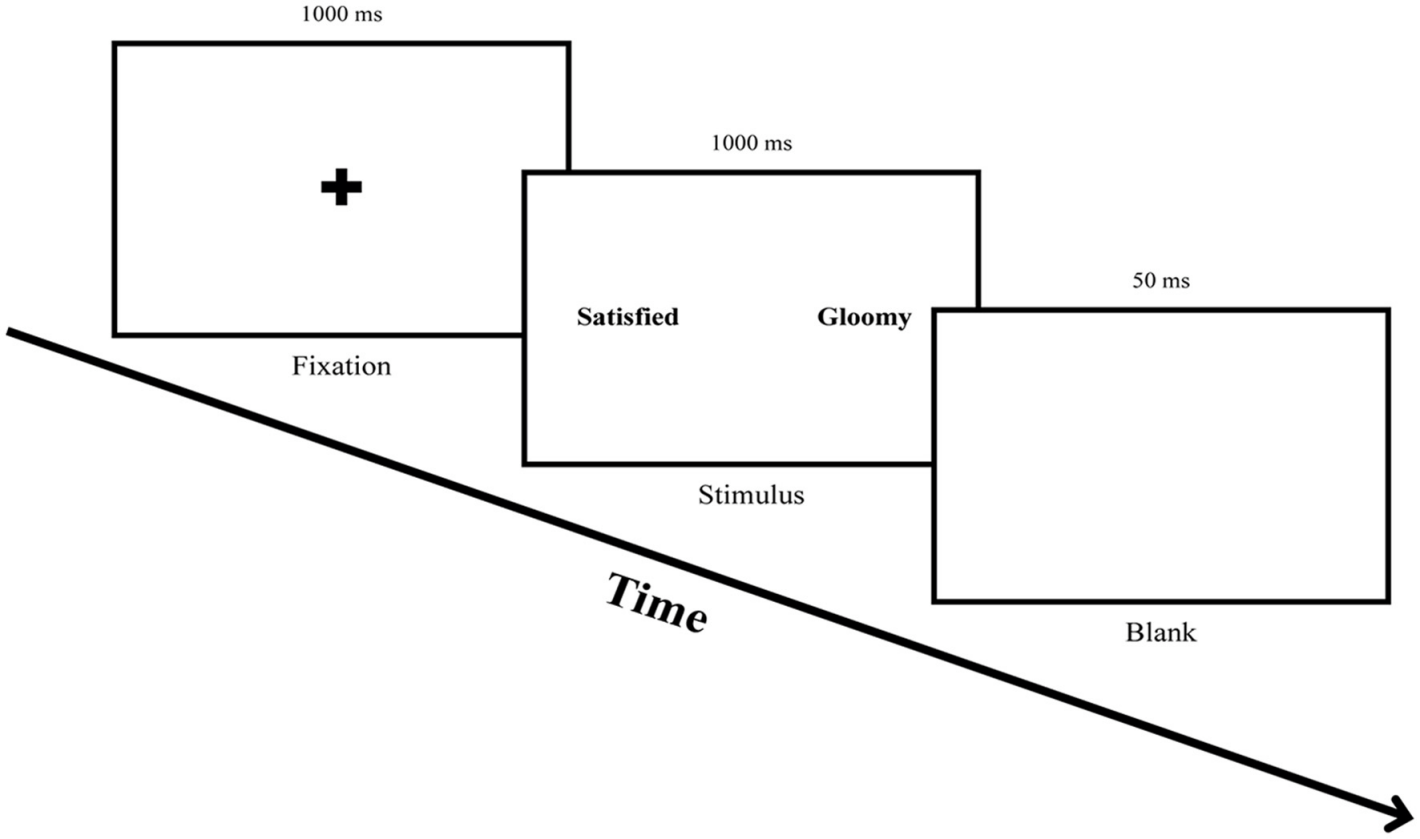
Procedure for the free-viewing task. Participants were asked to fix their eyes on the fixation cross for 1,000 ms and freely explore a word pair stimulus presented for 1,000 ms, followed by gazing at a blank screen for 50 ms.

To measure the attentional bias of participants before and after ABM, this study conducted a free-viewing task. The free-viewing task is beneficial for observing participants' natural eye movements since it records the eye movements while participants freely explored the presented stimuli (Ipata et al., 2006). Each trial started with a fixation cross in the center of the screen for 1,000 ms. An emotional word pair stimulus was presented for 1,000 ms after the fixation, followed by a blank screen for 50 ms (see Figure 2). Eight practice trials were followed by 60 trials of the main task.

There were three possible stimuli pair types, PW-NeuW, PW-DW, and DW-NeuW, and the locations of the words were counterbalanced to control the leftward bias. The word stimuli were matched for valence and arousal and presented side by side on a 21-inch monitor. The eye movement was measured by the iVew XTM Red-IV Eye Tracking System (SMI, Berlin, Germany) with a scanning rate of 60 Hz. The data on eye movements (percentage of initial fixation, latency to initial fixation, gaze dwell time) were recorded using BeGaze 3.1 software (SMI, Teltow, Germany). A fixation was defined as when the eye movements were stable for at least 80 ms within a visual angle of 1.4° (Armstrong et al., 2010).

#### 2.3.2. Attentional bias modification

Two types of ABM, such as positive ABM (pABM) and neutral ABM (nABM), in a form of a dot-probe task were conducted, and there were three possible stimuli pair types including PW-NeuW, PW-DW, and DW-NeuW. The pABM attempted to reduce the attentional bias of depression in terms of difficulty in disengaging from negative affective stimuli and encourage allocating attention toward positive affective stimuli. In 90% of the trials of pABM, a dot appeared at the same location as PW. The nABM was designed to have no modification effect. The composition of nABM was identical to pABM except for the location of the probe, which was equally presented in the location of PW, NeuW, and DW (Baert et al., 2010; Browning et al., 2012).

**Figure 3:**
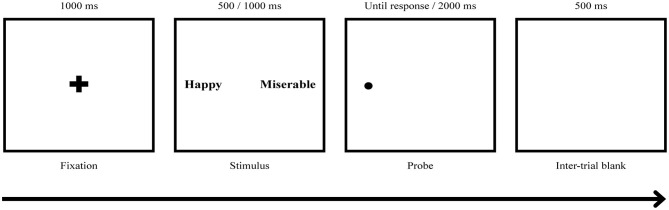
ABM task procedure. The participants responded to a dot probe displayed on the screen that appeared behind one of the words by pressing the key (“q” key for right and “p” key for left). Two types of ABM, positive ABM (pABM) and neutral ABM (nABM), were applied, and there were three possible stimuli pair types: PW-NW, PW-DW, and DW-NeuW. DW, Depression relevant negative affective words; NeuW, Neutral affective words; PW, Positive affective words.

Both ABMs were presented in two blocks with a 5-min short break given between the blocks. Each block consisted of 10 practice trials and 200 main trials, with 180 normal trials and 20 catch trials that provided animal word pairs as stimuli. A fixation cross was presented for 500 ms, followed by a word pair for 500 or 1,000 ms. Then, a small dot probe appeared behind one of the words, and the participants were instructed to press a response key (“q” key for right and “p” key for left) as quickly and accurately as possible to indicate the location of the probe. The probe remained on the screen until a response was made and disappeared after 2,000 ms (see Figure 3). The next trial started 500 ms after a response or disappearance of the probe. The location of the word and the order of stimuli presented including animal words for catch trials were randomized with an equal number of presentations. The ABM was programmed and presented using the E-prime version 1.0 (Psychology Software Tools, Inc., Sharpsburg, Pennsylvania, United States) and was displayed on a 21-in monitor.

### 2.4. Procedures

Screened participants by the scores of BDI-II and anxiety/somatization factor items of HDRS were divided into a mildly depressed group and a non-depressed group. The two groups were then randomly divided into two conditions (pABM and nABM). Participants were informed that the experiment was a study of arithmetic ability and attention to reduce demand effects. After obtaining informed consent, the experiment began with the initial free-viewing task. Participants were seated in front of the eye-tracking monitor, placing their heads on a chin rest located 60 cm from the monitor. Each word stimulus was sized to fit an 11.31° × 3.18° frame and the distance between the words was a visual angle of 15.19°. Participants were instructed to look at the screen, and stimuli were freely presented for 3 min.

After completing the initial free-viewing task, the participants performed a mental arithmetic task which is known for inducing psychological stress (Cacioppo et al., 1995). The participants were asked to mentally subtract 7 from 1,000 in a serial manner for 7 min as quickly and accurately as possible. They were asked to answer verbally, and if they gave the wrong answer, the experimenter provided feedback and the correct answer. Following the mental arithmetic task, participants were asked to report their feelings during the task by completing the Positive and Negative Affect Schedule (PANAS). A short break was provided for 3 min, and then the ABM was conducted. The size of the stimuli and the distance between participants and the monitor were similar to the free-viewing task. The post-session included another free-viewing task, a mental arithmetic task, and the self-report questionnaire administered in the same manner as previously described. Participants took part in the experiment for approximately 45 min and received a debriefing and a monetary reward of 10,000 Korean won (approximately USD$ 10).

### 2.5. Data analysis

Changes in the attentional bias between pre- and post-ABM training were analyzed using a 2 × 2 × 2 repeated measures ANOVA. Groups (mildly depressed group and non-depressed group) and types of ABM (pABM and nABM) were between-subjects factors, and session (pre-training and post-training) was a within-subjects factor. The dependent variable was the dwell time on each affective word stimulus, which indicated the degree of difficulty in disengaging from the stimulus. A paired *t*-test was conducted to test the effect of the within-subjects factor when there were significant interactions from the repeated measures ANOVA.

To analyze pre- and post-training session differences in emotional reactivity to stress, a 2 × 2 × 2 repeated measures ANOVA was conducted with group mildly depressed group and non-depressed group) and types of ABM (pABM and nABM) as between-subject factors and session (pre-training and post-training) as a within-subject factor. The dependent variables were the scores of PA and NA in PANAS following the mental arithmetic task. The analysis was conducted using SPSS 18.0 for Windows.

## 3. Results

### 3.1. Group characteristics

A total of 101 students successfully completed the entire procedure of the experiment; however, participants not recognized by the eye tracker were excluded, and data from a total of 78 participants were analyzed. The BDI scores were significantly different between the two groups, *t*_(76)_ = 9.657, *p* < 0.001. However, there was no significant difference within the groups, with all *p* > 0.1. There was no significant difference in the anxiety/somatization factor scores of the HDRS between groups, *t*_(76)_ = −0.378, *p* > 0.1, and within groups, with all *p* > 0.1. There were no significant differences in gender ratio between groups, $\chi_{\left(1\right)}^{2}$ = 1.260, *p* > 0.1, and within groups, with all *p* > 0.1. The group characteristics are presented in Table 2.

**Table 2:** Mean (SD) for the demographics and self-questionnaire scores of participants.

	**Positive ABM**		**Neutral ABM**	
	**Mildly depressed group**	**Non-depressed group**	**Mildly depressed group**	**Non-depressed group**
Age	22.35 (2.29)	22.17 (2.04)	22.61 (2.59)	23.42 (2.41)
Gender ratio (male/female)	3/20	5/13	6/12	6/13
BDI score	11.38 (3.45)	4.50 (2.88)	11.20 (3.37)	5.05 (2.85)
Anxiety/somatization factor score of HDRS	3.21 (1.89)	3.45 (1.28)	3.20 (1.74)	3.23 (1.41)

BDI, Beck Depression Inventory; HDRS, Hamilton Depression Rating Scale.

### 3.2. Self-questionnaires

With regards to the NA score from the PANAS, a three-way interaction among the group, type of ABM, and time was significant, *F*_(1, 74)_ = 19.935, *p* < 0.001, $\eta_{p}^{2}$ = 0.212. A two-way interaction between the group and dwell time was not significant, *F*_(1, 74)_ = 1.628, *p* > 0.1, $\eta_{p}^{2}$ = 0.022. However, two-way interactions between the type of ABM and time, *F*_(1, 74)_ = 14.454, *p* < 0.001 $\eta_{p}^{2}$ = 0.163, and the main effect of depression, *F*_(1, 74)_ = 12.431, *p* = 0.001, η*p*^2^ = 0.144, were significant. A paired *t*-test indicated significantly decreased scores of NA in the post-training session in the mildly depressed group with pABM, *t*_(22)_ = 5.562, *p* < 0.001, the non-depressed group with pABM, *t*_(17)_ = 6.102, *p* < 0.001, and the non-depressed group with nABM, *t*_(18)_ = 5.430, *p* < 0.001. Considering the interactions and follow-up test, the non-depressed group and the effect of pABM would cause the decrease of NA and the three-way interaction. The significant decrease of NA in the mildly depressed group with pABM supported the hypothesis that pABM would be related to the reduction of attention to depression-relevant negative stimuli and decreased emotional reactivity to stress after ABM of the current study.

Regarding the PA score from the PANAS, a three-way interaction among the group, type of ABM, and time was significant, *F*_(1, 74)_ = 5.780, *p* < 0.05, $\eta_{p}^{2}$ = 0.072. None of the two-way interactions were significant, with all *p* > 0.1. Only the main effect of the group was significant, *F*_(1, 74)_ = 12.431, *p* < 0.001, $\eta_{p}^{2}$ = 0.144. A paired *t*-test indicated that there was no substantial change in PA score among the mildly depressed group, with all *p* > 0.05. Non-depressed participants with pABM reported significantly decreased scores of PA, *t*_(17)_ = 2.378, *p* < 0.05; however, the non-depressed group with nABM exhibited no significant difference in PA scores in the post-training session, *t*_(18)_ = −0.175, *p* > 0.1. The significantly decreased scores of PA in the non-depressed group with pABM after the post-training session led to three-way interactions. The results indicated that pABM had neither a consistent effect on the non-depressed group nor the effectiveness of increasing dwell time on PA in the mildly depressed group.

### 3.3. Free-viewing task

Regarding the dwell time on DA, a three-way interaction among the group, type of ABM, and session was significant, *F*_(1, 74)_ = 3.86, *p* = 0.05, $\eta_{p}^{2}$ = 0.016. The significant three-way interaction included a significant two-way interaction between the group and session, *F*_(1, 74)_ = 5.64, *p* < 0.05, $\eta_{p}^{2}$ = 0.023. A follow-up test for the interaction between the group and session was conducted by a 2 × 2 repeated measures ANOVA depending on the type of ABM. In the pABM condition, the interaction between the session and the group was significant, *F*_(1, 74)_ = 9.02, *p* < 0.05, $\eta_{p}^{2}$ = 0.037. There were no significant effects of the condition of nABM, with all *p* > 0.1. A paired *t*-test was conducted to compare the dwell time differences between the pre- and post-training sessions. It indicated that the mildly depressed group with pABM attended significantly less to DW in the post-training session compared to that of the pre-training session, *t*_(22)_ = −3.57, *p* < 0.001. On the other hand, the mildly depressed group with nABM and the non-depressed group with pABM had no significant difference in the dwell time on DW after the ABM traning, with all *p* > 0.05 (see Table 3).

**Table 3:** Paired *t*-test results of dwell time differences between pre- and post-training depending on the group and type of ABM.

**Group**	**Types of ABM**	**Types of stimuli**	**Session**		***t***
			**Pre-training**	**Post-training**	
Mildly depressed group	Positive	DW	403.51 (228.28)	351.73 (188.76)	−3.57^*^
		PW	360.24 (249.00)	345.59 (253.19)	−0.77
		NeuW	344.81 (239.43)	367.12 (200.20)	1.41
	Neutral	DW	381.25 (200.06)	372.79 (187.98)	−0.55
		PW	358.80 (254.12)	333.60 (241.23)	−1.20
		NeuW	341.57 (239.55)	381.73 (186.35)	1.96
Non-Depressed group	Positive	DW	400.80 (207.38)	400.22 (207.83)	−0.04
		PW	418.44 (261.20)	403.33 (235.24)	0.82
		NeuW	354.83 (229.28)	369.73 (182.49)	−0.91
	Neutral	DW	380.58 (208.79)	367.09 (192.18)	1.79
		PW	384.61 (196.39)	426.67 (177.13)	−1.84
		NeuW	335.81 (128.06)	326.06 (121.19)	1.17

Positive, positive ABM; Neutral, neutral ABM; DW, depression-relevant negative affective words; PW, positive affective words; NeuW, neutral affective words;

* *p* < 0.001.

**Figure 4:**
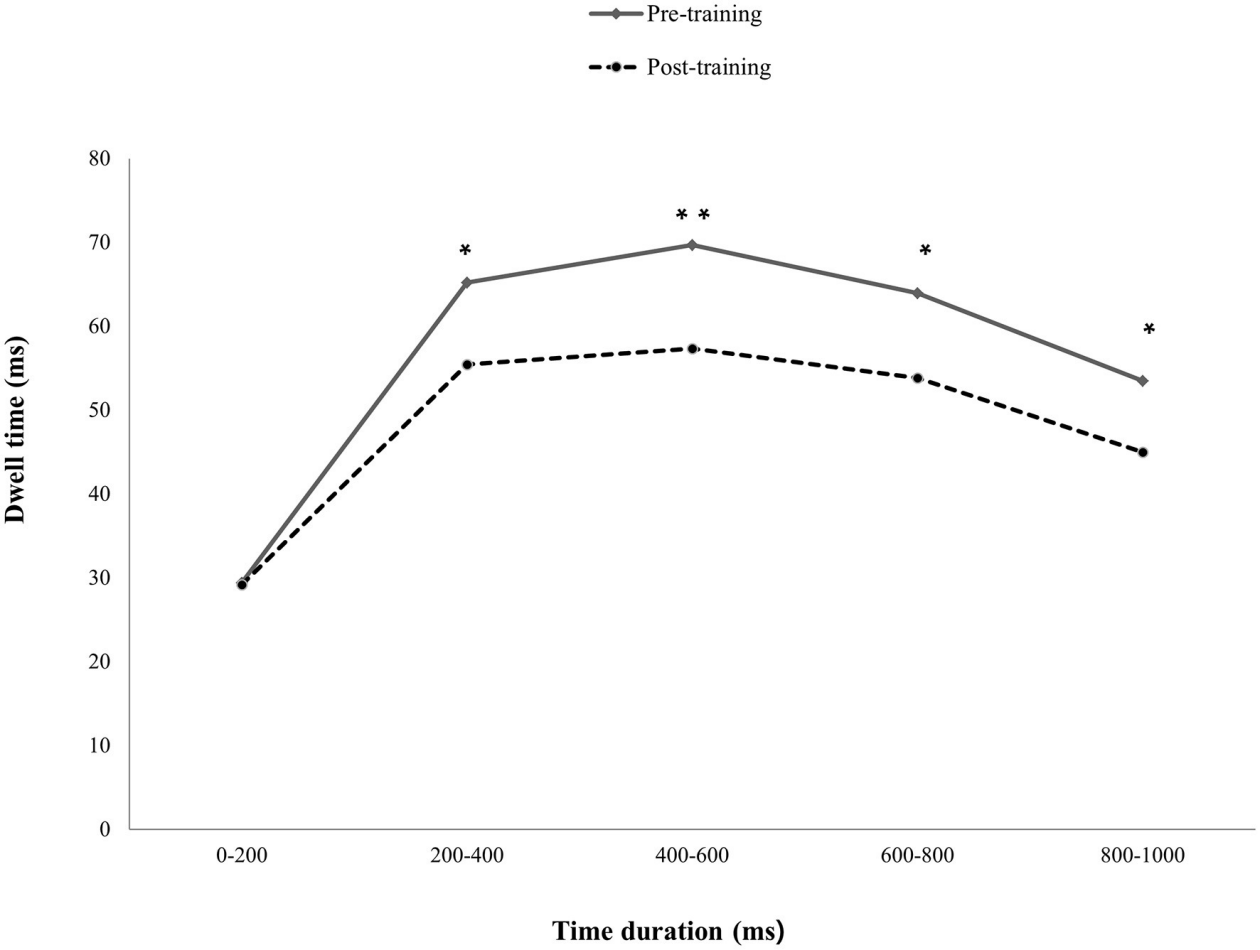
Differences in dwell time on DA between pre- and post-training sessions. The depressive participants had significantly decreased dwell time on depression-relevant negative affective words post-training compared to that of pre-training, except for 0–200 ms presentation time. ^*^p<0.05, ^**^p>0.01.

The results indicated that only the mildly depressed group with pABM had significantly decreased dwell time on DW after ABM training. Specifically, the total presentation time was divided into five-time courses (0–200, 200–400, 400–600, 600–800, and 800–1,000 ms). A paired *t*-test indicated that the dwell time was significantly decreased in the post-training session at 200–400 ms, *t*_(22)_ = 2.60, *p* < 0.05, 400–600 ms, *t*_(22)_ = 3.09, *p* < 0.05, 600–800 ms, *t*_(22)_ = 2.57, *p* < 0.05, and 800–1,000 ms, *t*_(22)_ = 0.2.38, *p* < 0.05. However, the change was not significant at 0–200 ms, *t*_(22)_ = 0.08 (see Figure 4). The results indicated that pABM was effective in reducing the difficulty in disengaging, which occurs after the evaluation of depression-relevant word stimuli as the current study expected. Specifically, ABM decreased the dwell time on emotional stimuli during the stimulus processing stage after 200 ms.

A three-way interaction among the group, types of ABM, and session was not significant with regard to the dwell time on PW, *F*_(1, 74)_ = 1.53, *p* > 0.1, $\eta_{p}^{2}$ = 0.001. Other effects were not significant as well, with all *p* > 0.1, except for the main effect of the group, *F*_(1, 74)_ = 23.59, *p* < 0.001, $\eta_{p}^{2}$ = 0.097. The results indicated that pABM did not increase dwell time on PW in either mildly depressed participants or the non-depressed group. Regarding dwell time on NeuW, a three-way interaction among the group, type of ABM, and time was not significant, *F*_(1, 74)_ = 1.82, *p* > 0.1, $\eta_{p}^{2}$ = 0.002. Except for the main effect of time, *F*_(1, 74)_ = 4.601, *p* < 0.05, $\eta_{p}^{2}$ = 0.019, other effects were not significant, with all *p* > 0.05. The results indicated that ABM had no significant effect on the dwell time on NeuW regardless of its type or the depressive status of participants.

## 4. Discussion

The current study aimed to verify the influence of ABM on the attentional bias of depression, defined as difficulty in disengaging from depression-relevant negative affective stimuli. The effects of ABM on depression have been examined in previous studies; however, the application has been limited in that the studies overlooked the fact that ABM intends to reduce the attentional bias in early attention (Leung et al., 2009), while the attentional bias of depression appears after the stage of evaluating stimuli in the later stage of attention. Additionally, this study expected the consecutive effect of decreased attentional bias by ABM on emotional reactivity to stress.

In the free-viewing task, mildly depressed participants with pABM dwelled significantly less on DW after pABM, and the result supported the hypothesis that pABM was effective in reducing the difficulty in disengaging, which occurs after the evaluation of depression-relevant word stimuli. This means that performing just two blocks (360 trials) of pABM was effective enough to reduce the difficulty in disengaging from DW in mildly depressed subjects. Regarding the significantly decreased attention to DW according to the time duration, pABM significantly decreased attention to DW after 200 ms, the attentional stage of evaluating stimuli and preparing the behavioral response, whereas there was no difference before 200 ms. The results supported the supposition that ABM may be more effective in training top-down control and enhancing general attentional control rather than just modifying attentional biases in the early stage of attention (Bar-Haim, 2010; Sallard et al., 2018; Li et al., 2023). In contrast to pABM, nABM did not enhance DW. This finding suggests that the training for searching positive stimulus rather than ignoring negative stimulus could play a pivotal role in ABM's efficacy (Waters et al., 2019). Our results were in line with the supposition; however, this might be due to the level of depression, as previous studies have reported effects on participants with mild depression, whereas participants with moderate or severe depression were unaffected by ABM (Baert et al., 2010). Therefore, ABM may be effective only in preventing the progression of depression in mildly depressed individuals because they do not exhibit their depressive symptoms apparently, while they have a high possibility of developing their depression because of attentional bias (Browning et al., 2012).

The results of the PANAS were in line with the results of the free-viewing task; therefore, it could be inferred that the reduced attentional bias by ABM brought further positive effects on emotional reactivity under stressful circumstances. Mildly depressed participants with pABM and non-depressed participants reported significantly decreased NA in the post-training session. It is worth noting that mildly depressed participants with pABM reported significantly decreased NA in the post-training session, while mildly depressed participants with nABM exhibited no significant change. This suggests that significantly decreased negative emotional reactivity to stress could be an effect of pABM; in other words, the negative emotional reactivity to stress could be weakened by ABM as it reduces the causal factor, attentional bias, in individuals exhibiting mild depression.

The current study has implications for applying ABM to depression and its potential therapeutic effects. The scope of previous studies was extensive to ascertain how ABM works and precisely what it targets because the pattern of attentional bias varies depending on the stages of attention (Leung et al., 2009). Thus, the study aimed to investigate which attentional stages are affected by ABM and the effects of ABM on its relevant symptoms (Lazarov et al., 2018; Klawohn et al., 2020). On this point, this study empirically supported that the mechanism of ABM can reduce the attentional bias of depression and its accompanying symptom, emotional reactivity to stress.

In addition, the results of the current study suggest the therapeutic advantages of ABM, indicating that it is an effective approach to reduce emotional reactivity to stress and obtain meaningful results. However, one of the drawbacks is that people with depression tend not to use mood regulation strategies by themselves (Joormann and Gotlib, 2007). By contrast, ABM does not encourage depressed participants to adopt unfamiliar strategies, while it has a remarkable effect on decreasing attentional bias and emotional reactivity to stress.

## 5. Limitations

This study had theoretical and practical implications; however, there were several limitations. First, this study identified evidence of enhanced disengagement ability from negative stimuli following ABM. Given the association between the dwell time on negative stimuli and the severity of depression shown in the previous study (Beevers et al., 2021), the results of this study could be interpreted as supporting the effectiveness of ABM. However, considering that the reduction of depressive symptoms after ABM was not verified, this study could not indicate that ABM has a direct effect on reducing depressive symptoms. Therefore, future studies need to replicate these findings through a longitudinal study to examine whether ABM reduced both the dwell time on negative stimuli and depressive symptom. Second, the findings from this study on individuals with mainly mild depression may not generalize to other age groups or people with severe depression, indicating limitations in the current study design. Third, this study had more female participants, introducing a potential bias in the results. This gender imbalance, especially with a relatively small number of male participants, can influence the generalizability of our findings. Future studies must ensure a balanced gender representation for broader applicability.

## 6. Conclusions

In conclusion, this study provided substantial evidence that ABM can effectively mitigate attentional bias in depression, particularly regarding the late stage of attention difficulty disengaging from depression-relevant negative stimuli. These findings highlight the potential of ABM as a tool for reducing attentional bias to negative stimuli and decreased emotional reactivity, which could be a crucial part of depressive symptoms. Also, to explain the mechanism and effectiveness of ABM in depression, future studies need to extend the application of ABM to the various levels of depression while also comparing the effects of ABM on the eye-movement patterns at each time duration.

## Data availability statement

The original contributions presented in the study are included in the article/supplementary material, further inquiries can be directed to the corresponding author.

## Ethics statement

The studies involving humans were approved by Chung-Ang University Institutional Review Board (No. 1041078-201405-HR-092-01). The studies were conducted in accordance with the local legislation and institutional requirements. The participants provided their written informed consent to participate in this study.

## Author contributions

SB: Conceptualization, Formal analysis, Methodology, Writing—original draft, review, and editing. SH: Formal analysis, Writing—review and editing. J-HL: Conceptualization, Formal analysis, Methodology, Writing—original draft, review, and editing.
